# Large Fæcal Abscess, Spontaneous Closure, Etc.

**Published:** 1886-02

**Authors:** 


### CULLINGS FROM CONTEMPORARIES.

"TRUTH is stranger than fiction." Many thing occur in medical and surgical practice which would sesm incredible were they not, often vouched for by the best authority; and most of these occurrences only seem to show how wonderful is that thing we call the *vis medicatrix naturæ*,—the recuperative and reparative powers of the human system.

Of all those wonderful occurrences, there are few, perhaps, which surpass that related in the *New York Medical Journal* for January 30th, by Dr. E. R. Chadbourne, of

### A LARGE FOECAL ABSCESS POINTING IN THE POPLITEAL SPACE AND RESULTING IN A SPONTANEOUS CLOSURE OF THE PERFORATED INTESTINE.

Mrs. ——, aged 37, (June 6, 1385), had been suffering from constipation, more or less. She complained of pain in the right lumbar region and hip, and the abdomen became very tympanitic. Enemata brought away only flatus. After a while an abscess formed—notwithstanding the bowels had been duly relieved, and pointing in the femoral region, opened internally and discharged its contents under the integument and facia of the thigh. The pus and fæces escaping, followed the course of the vastus externus muscle, and having no resistance except the loose cellular tissue, burroughed or gravitated down to the popliteal space, where it again pointed and was opened. The attending physicians made,also,openings in the thigh,which was tympanitic, and from which escaped flatus, pus and fæces. A drainage tube was inserted at the opening in the thigh and made to pass out at that in the popliteal space. The wound was properly treated, and in eight days the fistula in the bowel had closed, all discharge ceased and the patient recovered. The writer calls attention to a "peculiar idiosyncracy" of the patient to the toxic effect of iodoform, she having been poisoned by the local application of first, eighty grains, and a second time, by a smaller quantity. This is a mere coincidence, and has nothing to do with the strange fact of spontaneous closure of the fistulous opening in the bowel.

